# The integrated care pathway reduced the number of hospital days by half: a prospective comparative study of patients with acute hip fracture

**DOI:** 10.1186/1749-799X-1-3

**Published:** 2006-09-25

**Authors:** Lars-Eric Olsson, Jón Karlsson, Inger Ekman

**Affiliations:** 1 Sahlgrenska Academy at Göteborg University, Institute of Health and Care Sciences, Göteborg, Sweden; 2 Orthopaedic Department, Sahlgrenska University Hospital/Östra, Göteborg, Sweden

## Abstract

**Background:**

The incidence of hip fracture is expected to increase during the coming years, demanding greater resources and improved effectiveness on this group of patients. The aim of the present study was to evaluate the effectiveness of an integrated care pathway (ICP) in patients with an acute fracture of the hip.

**Methods:**

A nonrandomized prospective study comparing a consecutive series of patients treated by the conventional pathway to a newer intervention. 112 independently living patients aged 65 years or older admitted to the hospital with a hip fracture were consecutively selected. Exclusion criteria were pathological fracture and severe cognitive impairment. An ICP was developed with the intention of creating a care path with rapid pre-operative attention, increased continuity and an accelerated training programme based on the individual patient's prerequisites and was used as a guidance for each patient's tailored care in the intervention group (N = 56) The main outcome measure was the length of hospital stay. Secondary outcomes were the amount of time from the emergency room to the ward, to surgery and to first ambulation, as well as in-hospital complications and 30-day readmission rate.

**Results:**

The intervention group had a significantly shorter length of hospital stay (12.2 vs. 26.3 days; p < 0.000), a shorter time to first ambulation (41 vs. 49 h; p = 0.01), fewer pressure wounds (8 vs. 19; p = 0.02) and medical complications (5 vs. 14; p = 0.003) than the comparison group. No readmissions occurred within 30 days post-intervention in either group.

**Conclusion:**

Implementing an ICP for patients with a hip fracture was found to significantly reduce the length of hospital stay and improve the quality of care.

## Background

Hip fractures represent an increasing health problem in the Western world, mainly because the aging world's population. For instance, in the USA 350,000 incidents per year occur [1] and in the European Union 500,000 persons sustain a hip fracture per year [2]. In Sweden, the number of persons with a hip fracture is 18,000 and approximately 90% of them are over 65 years old, with more than half being octogenarians [3]. The incidence of hip fracture is expected to increase during the coming years [4], demanding greater resources and improved effectiveness on this group of patients. The challenge lies in efficient use of the limited resources available to provide a high quality care based on clinical evidence.

Changes in health status involve a process of transition [5]. Recovering from a hip fracture is a difficult period of transition, as the majority of elderlys live an independent life on the pre-morbid state while during the post-fracture period, they have to struggle in order to regain their well-being and pre-fracture functioning. Rehabilitation after a hip fracture requires a major effort from the patients, and other patients lost their independence [1,6,7]. Salkeld *et al*. [8] found that any loss of ability to live independently in the community has a considerable detrimental effect on an individual's perceived quality of life.

We have recently shown that despite age and health status, patients with a hip fracture had a strong will to recover, although they used different strategies to engage in the rehabilitation process [9]. These strategies must be identified by caregivers if successful rehabilitation is to be attained. In addition to the patient motivation, the care of patients with a hip fracture requires a team approach in which the co-ordination between the various aspects of care is important. Integrated care pathways (ICPs) have been proposed as one means of providing high quality care in a timely and cost-effective manner [10,11]. ICPs, which are used in many hospitals in several countries [12-16], have been described for over 45 conditions/procedures [17], including hip and knee replacement surgery and hip fractures. The degree to which they have succeeded in realising this potential for improving patient care has not been established, but there is enough supporting evidence to justify further research [17].

The aim of the present study was to evaluate the effectiveness of an ICP in patients with an acute fracture of the hip. The main outcome measure was the length of hospital stay; secondary outcomes were time from admission to the ward, operation, first ambulation, in-hospital complications and 30-day readmission. It was hypothesised that by coordinating and individualising the care path from admission to first ambulation and implementing a structured controlled training program it would be possible to reduce length of hospital stay and to decrease the number of medical complications.

## Methods

### Study design and procedures

A nonrandomized prospective study was conducted comparing an intervention group, guided by an ICP, with a comparison group, representing standard care [18]. The comparison group included 56 patients admitted to hospital between October 2003 and March 2004 and was compared with the intervention group by pre-fracture data regarding demographics, physical function and medical and mental status. The ICP was subsequently developed and implemented. All concerned personnel received special training and instructions for the successful implementation of an ICP. Data were then collected from 56 consecutive patients in the intervention group between October 2004 and March 2005. The patients received both oral and written information about the study at admission and informed consent was obtained from each patient. Participants in the study were only required to approve the use of their pre-fracture and clinical data. The study was approved by the human research ethics committee at the Medical Faculty, Göteborg University (Ö-420-03).

### Sample size

A previously conducted audit of hospital records of patients with a hip fracture indicated that the mean length of hospital stay was 31 days (SD 14.5) [19]. We estimated that 53 patients would be required in each group to achieve 80% power for detecting an 8-day reduction in length of hospital stay at a significance level of p < 0.05.

### Patient selection

Independently living ambulatory patients (with or without assistive devices) 65 years or older admitted to the hospital with an acute hip fracture were consecutively selected. Exclusion criteria were pathological fracture and severe cognitive impairment as assessed by the Short Portable Mental Status Questionnaire (SPMSQ) [20]. Approximately 35% of the patients in each group were excluded because of a low Pfeiffer test score. All eligible patients agreed to participate in the study. Three patients in the comparison group died before discharge from hospital.

### Data collection

All patients were interviewed by a nurse and demographic information was gathered on age, social status, type of living and degree of independence before the fracture using the Functional Recovery Scale (FRS) [21,22]. The physician who admitted the patient asked about co-morbidities and drugs while the interviewing nurse assessed the patients' nutritional status and symptoms of other potential problematic areas. At discharge, the patients' dependency on a walking aid and gait capacity was measured in order to determine their physical functioning upon leaving the hospital. Standard care consisted of a transferral system in which patients could be transferred to a geriatric department in hospital in order to facilitate post-operative rehabilitation. Decisions on which patients to transfer were made within the first few days after admission by an orthopaedic surgeon. Altogether, 28 patients were transferred.

#### The intervention

The intervention was developed with the intention of creating a care path with rapid pre-operative attention, increased continuity and an accelerated training programme without disturbing the flow of patients with other diagnoses. ICP documentation was developed in the form of a check list that displayed what to do and when to do it. Furthermore, the ICP covered all the critical elements of care and rehabilitation. The discharge plan, based on the patients' pre-fracture functioning, was developed within the first 48 h after admission. Long-term goals and intermediate goals were discussed with the patients' and their relatives in order to obtain a spirit of understanding. The nurses collaborated with the patients and their families throughout the hospital stay and were responsible in arranging contact with the communities' help service to secure the necessary training and support. As a part of the intervention, patients in the intervention group were transferred for medical reasons only and remained on the orthopaedic ward until they had attained an ADL level equivalent to their pre-fracture level, or until they did not progress further in their rehabilitation. No patient in the intervention group was transferred to other wards.

**Figure 1:**
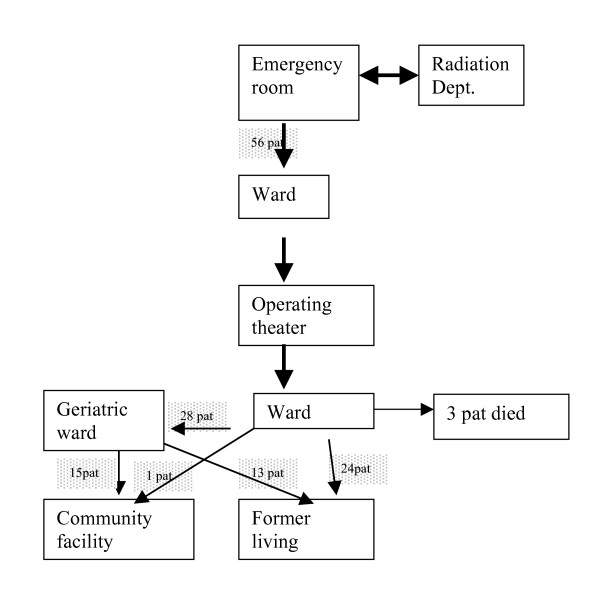
Comparison group. Clinical trajectory of care in the comparison group.

**Figure 2:**
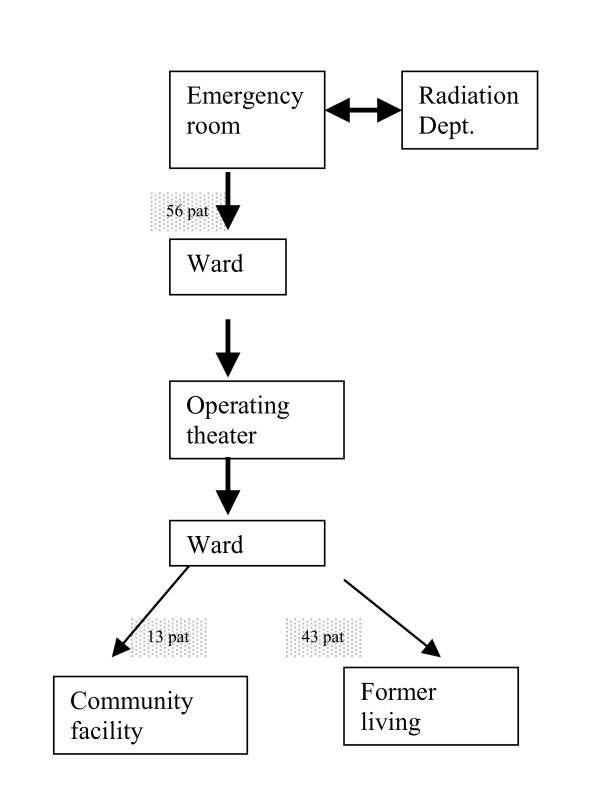
Intervention group. Clinical trajectory of care in the intervention group.

Before the start of the intervention, staff in the emergency room and radiology department was encouraged to attend and treat these patients rapidly so they could be admitted to the ward and prepared for surgery as soon as possible. Post-operatively, the earliest first ambulation was encouraged (if possible, the same day or the next morning). The training was then increased in accordance with the individual patient's prerequisites, although balancing between training and rest. Common rehabilitation interventions include providing advice, training, encouragement and listening to patients' concerns as well as drug treatment, physiotherapy, occupational therapy and help with use of appliances, equipment and daily living aids (Figures 1 and 2).

### Statistics

Parametric data were analysed with Student's t-test for independent groups while non-parametric data were analysed using Fisher's exact test and Chi-Square. Statistical significance was set to p < 0.05

## Results

The two study groups did not differ in any of the pre-fracture demographic variables (Table 1). The mean length of hospital stay was 26.3 days (SD 17.0) in the comparison group vs. 12.2 days (SD 3.5) in the intervention group (p < 0.000).

**Table 1:** Baseline data.

Data	Comparison N = 56	Intervention N = 56	P-value	Data	Comparison N = 56	Intervention N = 56	P-value
Female/male	42/14	41/15	1.0	Type of living			
Mean age	84	84		Flat	31	37	0.3
SD	(7.0)	(6.9)	0.9	House	13	7	
				Service flat	12	12	
Living							
With someone	19	14		Need of home help services			
Alone	37	42	0.4	None	34	28	0.4
				Once a week	7	9	
				Daily	15	19	
Place of accident				Type of walking aid			
At home	41	43	0.8	None	27	22	0.3
Outside home	15	13		Stick	11	8	
				Walking frame	18	26	
Number of co-morbidities				Gait capacity •			
Mean	2	3		Walking outdoors alone	31	26	0.4
Range	(0–8)	(0–8)	0.3	Walking outdoors with assistance	9	11	
				Walking indoors alone	13	13	
				Walking indoors with assistance	2	6	
General medical health†				Cognitive functioning at admission††			
A	10	5	0.1	Mean	8	7	0.4
B	33	29		Median	9	8	
C	13	22		Range	(3–10)	(3–10)	
Intra-capsular fracture	29	21	0.1	Pre-fracture independence†††•			
Hemiartroplasty	28	18		80 – 100 %	36	35	0.3
Osteosynthesis with	1	3		60 – 79 %	13	10	
Two parallel nails				< 60 %	6	11	
Extra capsular fracture	27	35		Mean	84	82	
				SD	(16.5)	(23.1)	

Data on pre-fracture and admission status are from the patients in both groups. There were no differences in pre-fracture demographics between the two groups.

• Missing data. Three patients died in the comparison group. Their available data were used.

† Ceder scale.

†† Pfeiffer's test.

††† Functional Recovery Scale.

Time spent at the different pre-operative ICP steps was measured and compared. The intervention group spent less time waiting at the emergency room before receiving care on the ward as compared with the control group (4 vs. 5 h; p = 0.02) and less time waiting for surgery (22 vs. 23 h; p = 0.6, ns)

The intervention group spent significantly less time between surgery to first ambulation (20 h vs. 28 h; p < 0.000), as well as from arrival to hospital to first ambulation (41 h, SD 13.2 vs. 49 h, SD 19.2; p = 0.01).

Contrary to the comparison group, significantly fewer patients in the intervention group developed complications: pressure wounds (8 vs. 19 patients; p = 0.02) and medical complications (5 vs. 14 patients; p = 0.003)

Data on physical functioning, ambulatory capacity and dependency on walking aids at discharge are shown in Tables 2 and 3. A non-significant difference in discharge destination was found in which 37 of the patients in the comparison group vs. 42 in the intervention group returned to their former place of residence (p = 0.517). There were no fracture-related readmissions within 30 days from discharge in either group.

**Table 2:** Physical functioning. The patients' physical functioning measured by basic-ADL pre-fracture and at discharge.

	***Pre-fracture***		***Discharge***		
B-ADL Level	Comparison *N = 56*	Intervention *N = 56*	Comparison *N = 53*•	Intervention *N = 56*	P-value
A	38	34	2	7	0.003
B	12	8	20	21	
C	2	6	5	8	
D	2	2	6	4	
E	-	-	3	7	
F	2	6	17	9	

• Missing data. Three patients died in the comparison group.

**Table 3:** Gait capacity. Use of walking aids and ambulation capacity at discharge.

Walking aid	Comparison *N = 53*	Intervention *N = 56*	P-value	Distance	Comparison *N = 53*	Intervention *N = 56*	P-value
None	1	0	0.02	10 meter	6	8	0.2
Walking stick	0	6		20 meter	4	4	
Walking frame	43	37		30 meter	10	7	
Walker	9	13		50 meter	3	11	
				100 meter	30	26	

## Discussion

The present study showed that our ICP was associated with a significantly shorter hospital stay, i.e. the number of care days was reduced by half compared with the comparison group. Despite a shorter hospital stay, the intervention group had better physical functioning and a higher ADL level. In the intervention group 25% more patients reached or approached their pre-fracture ADL level. Moreover, the intervention group was less dependent on walking aids, equal in gait capacity and more of the intervention patients returned to their former residence (Figures 1 and 2). These latter differences approached statistical significance and would likely have reached significance with a larger patient sample. A noteworthy fact was that it was possible to achieve this result without allowing a running-in period.

The randomised controlled trial design is considered the gold standard for evaluating interventions; however, its use in studies of this kind is somewhat problematic because such a design involves interactions between the patients and nurses. If two wards are used, it is difficult to know whether it was the change of actions or the interactions between the nurses and patients that contributed to any differences. In most studies a before-and-after design is preferred. The present study was carried out using a nonrandomized prospective design in which an intervention group was compared with a standard care group [18]. A disadvantage of this design is that it precludes conclusions regarding the true effects of an intervention, i.e. to know whether between-group differences are due to the intervention or to other factors. However, most studies of ICPs in patients with hip fractures have been conducted using this method [13-16].

The results of the present study are largely consistent with those reported in similar studies of ICPs in patients with a hip fracture [13,14,16,23]. In a controlled, prospective study Choong *et al*. [13] found that ICPs reduced the length of hospital stay without increasing the risk of complication or readmission rates. In another study Tarling *et al*. [16] noted that ICPs could reduce the length of hospital stay by 33%. Similarly, in a study comparing a fast track group to an ICP group Gholve *et al*. [14] found that ICPs could reduce the length of hospital stay by four days. On the other hand, Roberts *et al*. [15] found that whereas hospital stay increased, the quality of care was improved.

ICPs, which are designed to streamline and standardise various aspects of patient care, are structured multidisciplinary care protocols defining and specifying critical steps and progress in the care of various patient groups [24]. In implementing an ICP for acute hip fractures the most difficult component of the care trajectory in which to affect change are the steps from admission to first ambulation because so many different professionals are involved. Several studies have shown a correlation between waiting time for surgery and prolonged hospital stay [25,26], usually stating that more than 48 h of waiting will increase the hospital stay. In one study it was found that when the waiting time increased from 9 h to 16 h, the hospital stay increased by 19% [27]. It appears reasonable to keep the waiting time short because patient suffering can be relieved and precious time will be saved. For this reason, we made concerted efforts here and accomplished significant changes in two out of three outcomes. The continuity of caregivers and care content was maintained simply by eliminating transfers for other than medical reasons. Consequently, no transfers were made in the intervention group.

When the ICP protocol in the present study was developed, it was decided to build on the patients' engagement from an earlier study [9]. The hospital period is only the beginning of the rehabilitation process and it is important to facilitate a healthy transition process. In contrast to the care of younger patients, the care of elderly patients is more complex with more factors involved (such as health status, co-morbidities, motivation and cognitive functioning). It was believed that early improvements in the care path may start a positive chain reaction that can be kept going. An example is the earliest first ambulation that was planned either on the day of surgery or the next morning. Thus, the aim was to achieve a daily progress, which could be accomplished by being sensitive to the patients' resources (such as motivation) as well as being aware of physical limitations, i.e. ensuring a balance between training and rest. The early mobilisation and the strict training protocol reduced the number of pressure wounds. Moreover, the caregivers focused attention on each patient's status may have played a role in reducing the number of medical complications. In addition, early ambulation probably helped the patients to realise that they would be able to fully regain their ability to walk and thus their autonomy at an earlier stage.

## Conclusion

The ICP in the present study was effective. Although a significant reduction of time to first ambulation was achieved, the greatest effect was due to the care and attention given to the patients. Further investigation is needed to illuminate what components of the ICP are responsible for this reduction in care days and to determine whether there is an effect on one-year survival.

## Competing interests

The author(s) declare that they have no competing interests.

## Authors' contributions

LEO, JK, IE contributed to the development of the study protocol, design, data collection, statistical analysis, interpretation of data and preparation of the manuscript. All authors read and approved the final manuscript.
